# Prehabilitation: The underutilised weapon for chronic pain management

**DOI:** 10.1177/20494637241250239

**Published:** 2024-04-29

**Authors:** Lydia V. Tidmarsh, Richard Harrison, Katherine A. Finlay

**Affiliations:** 1 School of Psychology and Clinical Language Sciences, 6816 University of Reading, Reading, UK; 2 Centre for Integrative Neuroscience and Neurodynamics, 6816 University of Reading, Reading, UK

**Keywords:** Chronic pain, pain management, personalised care, prehabilitation, psychological preparation

## Abstract

**Objective:**

Prehabilitation encompasses preparatory clinical intervention(s) delivered during the period between diagnosis and treatment commencement. Despite widespread successful usage preoperatively, *psychological* prehabilitation is neglected in outpatient chronic pain management. Although pain management waitlists are associated with treatment attrition and psychological and physical decline, this time window is underutilised in preventing escalation. Waitlists present an under-explored opportunity to ‘prehabilitate’ patients waiting for treatment. This topical review aimed to: (1) examine the effectiveness of psychological prehabilitation for pain services; (2) evaluate the psychological and physical decline associated with waiting for pain management; (3) highlight key psychological prehabilitative targets for increasing treatment engagement; (4) promote pain management psychological prehabilitation within personalised pain medicine, building recommendations for future interventions.

**Methods:**

Studies regarding the impact of waitlists and prehabilitation for chronic pain were reviewed.

**Results:**

Findings demonstrated that the psychological constructs of patient expectations, health locus of control, self-efficacy and pain catastrophizing dynamically influence attrition, treatment engagement and outcomes while waiting. These constructs are amenable to change, emphasising their potential utility within a targeted waitlist intervention.

**Conclusions:**

Prehabilitating chronic pain patients towards treatment engagement could circumvent cycles of failed treatment seeking, preventing psychological and physical decline, and reducing healthcare utilisation. Utilising the waitlist to identify psychosocial risk factors (external health locus of control, low self-efficacy and high pain catastrophizing) would identify *who* requires additional support to prevent increased risk of treatment failure, enhancing personalised care before prescribed treatment is accessed. This review cements the urgent need for pain services to engage proactively with prehabilitation innovation.

## Introduction

Chronic pain presents an increasing global public health issue, impacting an estimated 20% of adults worldwide.^1,2^ Societal costs attributed to chronic pain are high, ranging from $560 to $635 billion due to pain and pain-related comorbidity care provision, and days lost to productivity.^3^ Moreover, economic and service strains have dramatically increased in recent years; vast backlogs due to withdrawn treatment during the COVID-19 outbreak has resulted in extensive waitlists.^4^ Furthermore, chronic pain is a prevalent symptom of long-COVID,^5^ contributing an additional subset of patients waiting for treatment. Chronic pain is highly associated with psychological comorbidities, that together, entrench pain and detrimental psychological decline over time.^6,7^ The longer patients are left waiting, unfurnished with psychological strategies, the greater the cumulative detrimental physical and psychological impact.^8^ Pain and depression interact bidirectionally^9^ with a dose-dependent relationship; greater pain intensity is associated with elevated levels of depression.^6^ Thus, intervening at the earliest point to prevent escalation is intuitive to reduce health, social and economic burdens. At present, there is an underutilised weapon in chronic pain management that is used pre-operatively in other physical conditions: *psychological prehabilitation*.

Prehabilitation is the clinical intervention between diagnosis and commencement of treatment.^10^ This aims to promote physical and psychological wellbeing to prevent or reduce the severity of future complications. Evidence shows better general preoperative health is associated with improved postoperative outcomes across a range of chronic conditions including musculoskeletal disorders, chronic pelvic pain and cancer.^11–13^ Prehabilitation approaches are largely applied within preoperative settings, an example being Enhanced Recovery After Surgery (ERAS).^14^ Traditionally, they are bimodal, encompassing physical exercise and nutritional optimisation.^15^ However, the complex relationship between chronic pain and psychology requires greater emphasis on psychological elements in the pre-treatment phase.^16,17^ Hence*, trimodal prehabilitation* additionally includes psychological strategies.^18^ The chronicity of persistent pain requires patient active participation in self-managing their condition.^19,20^ To do so, individuals need to be equipped with successful self-management strategies at the earliest point. However, in practice, psychology is not currently strongly integrated at *pre-treatment* in outpatient settings. Thus, the aim of this review is four-fold:

(1) To examine the effectiveness of psychological prehabilitation for pain secondary care;
(2) To evaluate psychological and physical decline associated with long treatment delays;
(3) To highlight prehabilitative psychological targets to prevent patient decline and increase engagement;
(4) To promote psychological prehabilitation for pain management within personalised pain medicine, building recommendations for future prehabilitation research.

## Why psychological prehabilitation?

Prehabilitative strategies are highly cost effective; pre-operative ERAS protocols indicate a mean saving of $1458.62 per patient, 21.5% of the total cost of surgical procedures from reduced subsequent healthcare utilisation.^21^ Psychological intervention is utilised as a chronic pain management strategy (at point of treatment) due to its efficacy and cost effectiveness.^22^ Thus, on an economic level, exploring psychological *prehabilitation* for chronic pain self-management is valuable to further enhance the cost benefits of pre-intervention protocols. Unsupported long treatment delay increases attrition in pain management.^23^ Thus, clearly, without psychological preparation, patients are less likely to be motivated to engage in treatment once accessed. As motivation and capability beliefs determine engagement in exercise participation^24,25^ and diet,^26^ psychological prehabilitation will also have indirect benefits of adherence to the other trimodal prehabilitation elements. Behavioural decisions are continuously required; whether to participate in prehabilitation *at all*, and thereafter, *daily* to upkeep pain management strategies, physiotherapy and nutritional regimes.^24^ Thus, psychology functions as the lever that facilitates adherence, increases interest, and initiates reflective motivation over *why* self-management is important. Therefore, psychological prehabilitation must be reasserted as a critical pre-requisite for pain management to optimise treatment.

Where few trimodal prehabilitation strategies have been implemented pre-operatively, psychological elements include cognitive behavioural strategies, preoperative education, behavioural instruction and stress management to influence pain perception and psychological wellbeing.^12^ Postoperative pain, behavioural recovery, affect and healthcare utilisation are reduced as a result of psychological prehabilitation.^12,18,27^ Implementing education within chronic pain interventions is also found to increase internal locus of control, pain self-efficacy, positive perceptions, life satisfaction and reduce pain-related interference, pain intensity and anxiety.^18,28,29^ Anxiety and depression are also significantly reduced following trimodal prehabilitation utilising psychological strategies in chronic pain patients waiting for surgery.^30,31^ Given such results for pre-surgical psychological prehabilitation, it is likely these benefits may also be extended to prehabilitation before secondary care.

## Psychological and physical decline: The need for waitlist optimisation

Consistent excessive strain from under-resourced health services globally has resulted in elongated waitlists.^4^ These have been further exacerbated by the COVID-19 outbreak^32^ reflected in a 46.3% increase in people waiting for NHS treatment in the UK; 6.48 million people as of April 2022, compared to 4.43 million in February 2020 (pre-pandemic).^4^ Globally, average Pain Management Programme (PMP) waiting time ranges from 7.9 months (Canada)^33^ to 2-years (UK).^34^ Even prior to the added service pressures induced by the COVID-19 pandemic, these waiting times drastically exceed the International Association for the Study of Pain’s (IASP) guidelines of a 2-month wait-time for routine conditions, and 1 month for urgent or semi-urgent cases. Thus, the need for waitlist optimisation is rising.

The rapidity of treatment delivery for people with chronic pain is paramount for managing psychological vulnerability. Chronic pain is an independent predictor of higher suicide likelihood.^35^ Furthermore, comorbid anxiety and chronic pain are also associated with greater odds of suicidal ideation and attempts.^36^ Comorbid anxiety and chronic pain is well established^35–37^; a nationally representative sample indicating more than 60% of patients with generalised anxiety disorder had at least one chronic pain condition.^36^ Extensive waitlists are characterised by anxiety and uncertainty,^38^ and patients are left feeling helpless, disregarded and lost within the system.^39,40^ Reduced treatment as a result of COVID-19 led to worsening pain, greater stress, anxiety and depression.^8,41,42^ Concerningly, empirical data suggests rapid psychological decline occurs within 5-weeks on waiting lists, depleting health-related quality of life.^7^ Therefore, intervention to support people living with pain during their waiting time is critical for psychological health and to prevent early mortality or suicidality.

Alongside psychological decline reduced physical health during long waitlists is also observed. Deterioration of pain-related conditions, increased pain intensity, fatigue, limited mobility and reduced activity engagement are all associated with longer waiting times.^40^ Indeed, 33%–65% of chronic pain patients report worsened pain-related symptoms and functional disability when on extensive waitlists.^43,44^ Permanent disability benefits and unemployment are also increased with elongated treatment delay.^33,44^ Physical decline further exacerbates psychological distress, elevating anxiety and frustration.^40^ This creates a cumulative effect between physical and psychological outcomes during longer waitlists. Notably, concurrent physical and psychological decline are observed in pain patients waiting for longer than 6 months.^40^ Thus, clearly, in the context of increased service pressure, waitlists represent a substantial challenge for patients and a ‘critical time window’ for prehabilitation.

## Psychological targets within prehabilitation to increase engagement

**Figure 1.**
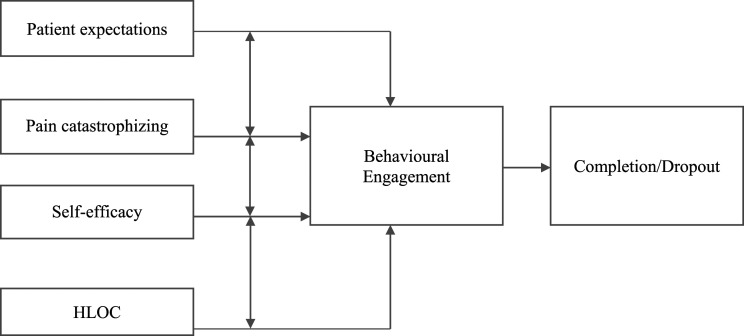
The interrelating psychological constructs influencing behavioural engagement for pain management. *Note.* Patient expectations, pain catastrophizing, self-efficacy and health locus of control (HLOC) are all independently associated with behavioural engagement with self-management and treatment completion or attrition.^23,39,65,81,82^ Negative expectations are associated with higher pain catastrophizing.^57^ Pain catastrophizing has a negative relationship with self-efficacy; higher levels of pain catastrophizing are negatively correlated with lower self-efficacy.^109^ Self-efficacy and health-related locus of control are directly associated 46; high levels of internal HLOC are positively correlated with high pain self-efficacy, having greater behavioural engagement in pain self-management strategies.^47^

To effectively optimise the pre-treatment period, it is important to identify factors influencing behavioural engagement with treatment.^45^ Evidence suggests these include interacting factors of patient expectations, health locus of control, self-efficacy and pain catastrophizing (*see* Figure 1).^40,46–48^ Moreover, the Faculty of Pain Medicine^49^ recommends earlier application of pain management principles, and these constructs are central to the British Pain Society^50^ guidelines for PMP content; such cognitive elements of perceived behavioural control are highly relevant to pain self-management engagement. Encouragingly, these factors are flexible to change through targeted intervention utilising cognitive behavioural and acceptance and commitment strategies, indicating their value in prehabilitation.

## Patient expectations

Patient expectations are defined as the set of beliefs regarding anticipated treatment and its potential effects^51^; these are predictive of treatment outcomes.^52,53^ Positive expectations, compared to negative, are associated with greater improvements in disability, functional activity and reduced pain intensity, sustained beyond 6 months.^54–56^ Negative expectations of treatment and symptom worsening are associated with reported increased pain severity up to 8 months later.^44^ In osteoarthritis patients, those with moderate to high expectations prior to treatment reported improved pain intensity, self-efficacy, quality of life, pain catastrophizing, and reduced use of pain medication following psychological intervention.^57^ Therefore, even moderate, and not necessarily *high,* expectations can improve pain management outcomes.

Long waiting times with no information may also lower expectations regarding service quality and treatment outcomes.^40^ Lack of information can lead to feelings of abandonment and disenchantment.^40^ Thus, provision of sufficient information can be a simple way of abating a cascade of negative cognition and reduced engagement. Elongated waitlists pose a barrier in of themselves to accessing treatment; recent evidence suggests waiting lists of 4 months or longer increase the risk of not attending the first PMP session by 25%.^23^ Thus, improving patient expectations through increased communication whilst waiting would likely increase perceived support, improving expectations and thus accessibility.

Managing patient expectations of potential *outcomes* is especially important prior to accessing treatment for chronic pain. While PMPs are effective in improving pain self-efficacy and pain interference, pain *intensity* may not significantly reduce.^58^ When patients are left with inaccurate outcome expectations during extensive waitlists from incomplete information provision, this leads to extreme dissatisfaction once treatment is accessed.^40^ When treatment outcomes are not congruent with patient expectations, frustration ensues, resulting in dropout.^59^ This would further compound the increased susceptibility to attrition associated with long waitlists,^23^ and short circuit a cycle of failed attempts and excessive healthcare utilisation. Therefore, targeting expectations of treatment outcomes via pain education during waitlists may improve both emotional wellbeing and completion rates. Consequently, this could reduce financial and service strains at a systemic level.

## Health locus of control

An individual’s belief of their capability to exert control over their condition is fundamental to successful pain self-management.^60–62^ Health-related Locus of Control (HLOC) is a central construct conceptualised as either: (1) Internal (patients hold control and influence over their health), (2) Powerful Others (doctors and healthcare professionals hold greatest influence over health), and (3) Chance (health is determined by fate or chance events outside of individual control).^63^ Within behavioural science, capability is one of three interacting elements (capability, opportunity and motivation) predicting behavioural change (COM-B model).^45^ For a desired behaviour to occur, these behavioural elements must be supported.^64^ Thus, given the integral role of perceived capability within HLOC, it is likely a fundamental construct for increasing pain self-management engagement.

Evidence suggests higher levels of internal HLOC are associated with greater competence and engagement in pain self-management for chronic disorders.^63,65^ Systematic review evidence indicates that HLOC is predictive in determining rehabilitation outcomes in chronic pain, with internal HLOC associated with optimal improvement.^46^ Higher internal HLOC at *pre-treatment* significantly predicts greater reductions in pain intensity following multidisciplinary intervention, compared to those with low internal HLOC.^66^ Greater functional improvement is also observed in patients with higher internal HLOC at pre-intervention, in both surgical (18 weeks post operatively),^67^ and multidisciplinary settings.^66,68^ As chronic pain patients tend to present greater levels of external HLOC (69% identified as external HLOC vs 31% as internal)^48^; optimising personal agency and belief towards capacity for self-management represents an important intervention objective. HLOC is amenable to change; CBT-based self-management interventions display efficacy in increasing perceived control immediately post-intervention and internal locus of control 12 months later.^23^ Together, this evidence highlights the importance of HLOC at pre-intervention and its malleability. Thus, it is only efficient to implement such targets within prehabilitation to improve both treatment engagement and outcomes.

## Self-efficacy

In the context of health, self-efficacy is defined as an individual’s belief in their ability to engage in health management behaviours pertinent to their condition.^67^ Whilst patients are waiting, identifying those with low self-efficacy would enable stratification to additional support to prevent increased risk of treatment failure. Higher levels of pain self-efficacy encompass the perception of sufficient internal resources to overcome pain-related demands, and thus the ability to effectively cope with pain.^69^ Pain self-efficacy is a greater independent predictor of disability, above pain severity,^70^ and a key mediator of behavioural change.^69,71^ Patient engagement in pain self-management programmes is greater in those with higher pain self-efficacy^72^ and low pain self-efficacy is a barrier to accessing treatment.^39^ High self-efficacy is also associated with positive affect and greater physical function.^73^ In chronic pain patients, greater levels of pain self-efficacy are protective of psychological health^74^: (1) they moderate the direct effects of pain intensity on depression; and (2) indirectly lower levels of depression and pain by reducing pain catastrophizing.^75,76^ Importantly for intervention, self-efficacy is a construct flexible to change; pain self-management interventions are effective in increasing self-efficacy, improved physical function, reduced pain intensity and depression.^72,75–77^ Thus, when considering the psychological decline associated with long waitlists,^7,8^ enhancing self-efficacy prior to treatment is an insightful psychological prehabilitative strategy in priming patients for engagement once treatment is accessed.^78–80^

## Pain catastrophizing

Screening for pain catastrophizing (PC) during the waiting list provides a key opportunity to stratify patients at-risk of worsening pain. For people living with persistent pain, cognitive rumination, catastrophic thinking and helplessness can develop, increasing pain intensity and interference.^73,81^ These are interacting dimensions of PC: rumination (negative preoccupation with pain and pain-related fears and thoughts); magnification (the amplification of the salience of pain on one’s health); and helplessness (perceived despair regarding the ability to control the pain experience).^82^ Furthermore, evidence shows higher PC at pre-intervention significantly predicts lower engagement in PMPs^81^ and attrition.^82^ As PC is typically elevated during waitlists,^7^ and more broadly, as attrition rates for PMPs are generally high (up to 51%),^83^ targeting PC during the waitlist may activate this period of decline to improve PMP completion. Identifying patients with higher PC at point of triage would facilitate optimal implementation of further support, reducing attrition and subsequent healthcare utilisation.

Importantly, PC is amenable to change through psychological intervention.^84^ Strategies including pain science education, exercise for pain self-management^85^ and cognitive behavioural therapy display reduced levels of PC post-intervention.^86^ Moreover, improved PC is also related to greater benefits following multidisciplinary pain management including greater reductions in pain intensity, interference, depression and functional disability by 30%.^84,87^ Due to the influence of PC on treatment engagement,^81^ completion^82^ and pain-related outcomes,^88^ together, this suggests reducing PC as an effective strategy for prehabilitation efforts within clinical practice.

## Clinical recommendations: Prehabilitation within personalised medicine

Psychological prehabilitation is critical in targeting the psychological processes involved in the exacerbation of chronic pain. Thus, utilising the waitlist period for patient stratification according to these psychosocial risk factors (expectations, health locus of control, self-efficacy and pain catastrophizing) within prehabilitation may ultimately enhance treatment retention and PMP completion.^52,89^ This would provide understanding for *whom* further support is best directed towards; the patient would receive personalised care before treatment is even accessed. This predictive assessment could be applied easily, simply via the application of widely available psychometrics. The need for personalised pain treatment based on patient characteristics is increasingly recognised.^90–92^ This cultural clinical shift, led by IASP, emphasises the difference between personalised and stratified care.^90^ Stratified care is cost effective,^92^ however, personalised care increases patient satisfaction, re-centring clinical focus to that of the gold-standard person-centred approach.^93^ Personalised care acknowledges individual differences even within stratified subgroups, taking into consideration the patient’s values and perspective. This encompasses reformulating maladaptive cognitions and beliefs and considering the influence of self-efficacy when determining treatment pathways.^90^ Importantly, chronic pain patients express a desire for personalised treatment and self-management strategies.^93^ Given that patient expectations are a predominant factor for patient engagement, this would have great influence in reversing the negative cascade to attrition. By improving retention and completion, such psychologically focused pre-treatment intervention would improve healthcare provision on a macro-scale, reducing service and economic burdens in the long-term.

## Viable implementation modalities for prehabilitation

Currently, there is no consensus on the best modality for psychological prehabilitation delivery for outpatient chronic pain. There is the risk that while psychological prehabilitation could be valuable, if it is not employed efficiently, it may lead to an additional waitlist for starting prehabilitation itself. However, evidence from surgical interventions suggests that multiple digital modalities are effective.^94,95^ Pre-surgical digital interventions vary widely in their content; however, they typically include tailored goal-setting, education, cognitive behavioural principles, reminders, activity and sleep logs.^95^ An online, self-guided mindfulness-based stress reduction (MBSR) programme encompassing a total of 16 hours audio-video content, comprising 8 primary sessions and 6 additional hours, resulted in significantly reduced pain 30 days after surgery.^96^ Lower pain interference, disability and greater physical function was also observed 3 months post-surgery, with mindfulness identified as a predictor of change in physical function^96^ and pain interference at 12 months.^94^ A self-guided internet-delivered pain-coping skills training also increased self-efficacy for pain management compared to standard controls.^97^ In patients with chronic conditions undergoing surgery, a combination of psychoeducation via information booklets and diary keeping significantly reduced postoperative pain compared to standard care.^98^ Psychoeducation provided through websites is also superior in improving knowledge and satisfaction regarding pending treatment, compared to a surgical consultation alone.^99^ Accessibility, convenience, self-monitoring and progress reports are all key benefits reported by patients undertaking digital interventions while waiting for surgery.^95^ Psychological digital prehabilitation strategies can either be guided by a psychologist, or self-guided via watching video content. Each will have different requirement of resources, yet self-guided may be more appropriate for reducing implications on additional waitlists as it will not depend on staff availability to lead the sessions. Thus, due to the effectiveness and lower staff resource requirement, self-guided online psychological intervention may be an efficient implementation style for psychological prehabilitation in outpatient chronic pain. Regarding digital modality, there is greater engagement with mobile apps and Facebook community group pages compared to websites, due to push notifications and reminder functions.^100,101^ Therefore, perhaps the development of an app comprising education and self-guided cognitive behavioural principles would be effective for an outpatient chronic pain prehabilitation intervention. Such promising evidence for digitally implemented pre-surgical prehabilitation suggests value in exploring such.

## Future directions

At present, the evidence base for chronic pain prehabilitation grounded in psychological theory and behavioural science is lacking.^102^ Research to develop an innovative prehabilitation intervention to activate the waitlist for pain self-management is required. For maximum impact at an individual and systemic level, it is important such intervention design is grounded in behavioural science.^46^ The Behaviour Change Wheel (BCW)^46^ provides a structured approach for intervention development,^103^ greatly utilised within health policy implementations. Systematic review and meta-analytic evidence suggest rehabilitation practice, with a behaviour change focus, is effective in changing physical activity and eating behaviours in cancer patients.^104^ However, no guidelines currently exist for PMP *prehabilitation,* encompassing patient preferred content, recommendations for delivery, or knowledge of the prospective influence on patient satisfaction.^105^ To identify pathways for waitlist intervention design, further research is needed exploring the facilitators and barriers to engagement from the patient perspective, strategically mapped to the COM-B model. Patient involvement is crucial for effective intervention design, as aligned with NICE guidelines of gold-standard care.^98^ Thereafter, the Behaviour Change Technique Taxonomy Version 1 (BCTv1)^46^ can be applied to identify Behaviour Change Techniques (BCTs) to target specific processes to initiate behavioural action. BCTs including ‘self-monitoring’, ‘instruction on how to perform a behaviour’ and ‘behavioural practice’ effectively increase physical activity adherence in chronic pain patients.^102,106^ Therefore, this gap needs to be addressed for *prehabilitative* practice. To rectify inconsistent reporting of behaviour change interventions within literature,^45^ the Behaviour Change Intervention Ontology (BCIO),^107^ together with the BCIO data extraction template,^107^ should be utilised as a comprehensive and systematic framework for high quality reporting of BCIs and their contexts. Doing so will enable the development of an effective, replicable, theoretically grounded prehabilitation waitlist intervention.

## Conclusion

Innovative psychological prehabilitation offers a valuable enhancement to current outpatient pain practice. The wholly negative impacts of long treatment delay can be reinterpreted as presenting an underutilised opportunity to target barriers of self-management engagement. Psychological constructs of positive expectations, internal health locus of control, high self-efficacy and low pain catastrophizing all improve pain outcomes and behavioural engagement.^47,52,58,108^ Critically, these psychological factors are all amenable to change through psychological intervention.^23,52,76^ To avoid the risk of creating additional waitlists to start prehabilitation itself, digital modalities involving self-guided cognitive behavioural principles and education suggests promise for an effective and efficient implementation style.^96,97^ Activating the waitlist by intervening during this period, utilising behavioural science principles, could reverse the downward spiral of pain, negative affect and maladaptive cognitions presenting during the waiting period. Health and social care systems could improve efficiency by priming patients for treatment engagement, reducing repeat cycling through failed treatment attempts and excessive healthcare utilisation. Moreover, phenotypically identifying patients with increased susceptibility to treatment failure within the *waitlist* would enable stratified treatment pathways and enhanced personalised care at the earliest point, optimising treatment outcomes. Significant potential exists to extend psychologically-led prehabilitation intervention beyond PMPs, with application to other clinical waiting lists. Further research is needed to develop such prehabilitation practice which can be applied within health settings worldwide, improving healthcare globally.

## References

[1] Eccleston C . Chronic pain as embodied defence: implications for current and future psychological treatments. Pain 2018; 159(Suppl 1): S17–S23. 30113943 10.1097/j.pain.0000000000001286

[2] Rice ASC Smith BH Blyth FM . Pain and the global burden of disease. Pain 2016; 157: 791–796. 26670465 10.1097/j.pain.0000000000000454

[3] Gaskin DJ Richard P . The economic costs of pain in the United States. J Pain 2012; 13: 715–724. 22607834 10.1016/j.jpain.2012.03.009

[4] BMA NHS Backlog Data Analysis. British Medical Association. 2023. https://www.bma.org.uk/advice-and-support/nhs-delivery-and-workforce/pressures/nhs-backlog-data-analysis. (accessed 30 October 2023).

[5] Calvache-Mateo A López-López L Martín-Núñez J , et al. Pain and clinical presentation: a cross-sectional study of patients with new-onset chronic pain in long-COVID-19 syndrome. Int J Environ Res Publ Health 2023; 20: 4049. 10.3390/ijerph20054049 PMC10001485 36901059

[6] Angst F Benz T Lehmann S , et al. Extended overview of the longitudinal pain-depression association: a comparison of six cohorts treated for specific chronic pain conditions. J Affect Disord 2020; 273: 508–516. 32560947 10.1016/j.jad.2020.05.044

[7] Lynch ME Campbell F Clark AJ , et al. A systematic review of the effect of waiting for treatment for chronic pain. Pain 2008; 136: 97–116. 17707589 10.1016/j.pain.2007.06.018

[8] Eccleston C Blyth FM Dear BF , et al. Managing patients with chronic pain during the COVID-19 outbreak: considerations for the rapid introduction of remotely supported (eHealth) pain management services. Pain 2020; 161: 889–893. 32251203 10.1097/j.pain.0000000000001885 PMC7172975

[9] Viana MC Lim CCW Garcia Pereira F , et al. Previous mental disorders and subsequent onset of chronic back or neck pain: Findings from 19 countries. J Pain 2018; 19: 99–110. 29031785 10.1016/j.jpain.2017.08.011 PMC6839827

[10] Silver JK Baima J . Cancer prehabilitation: an opportunity to decrease treatment-related morbidity, increase cancer treatment options, and improve physical and psychological health outcomes. Am J Phys Med Rehabil 2013; 92: 715–727. 23756434 10.1097/PHM.0b013e31829b4afe

[11] Carli F Gillis C Scheede-Bergdahl C . Promoting a culture of prehabilitation for the surgical cancer patient. Acta Oncol 2017; 56: 128–133. 28067101 10.1080/0284186X.2016.1266081

[12] Powell R Scott NW Manyande A , et al. Psychological preparation and postoperative outcomes for adults undergoing surgery under general anaesthesia. Cochrane Database Syst Rev 2016; 2016: CD008646. 27228096 10.1002/14651858.CD008646.pub2 PMC8687603

[13] Santa Mina D Scheede-Bergdahl C Gillis C , et al. Optimization of surgical outcomes with prehabilitation. Appl Physiol Nutr Metabol 2015; 40: 966–969. 10.1139/apnm-2015-0084 26300015

[14] Dietz N Sharma M Adams S , et al. Enhanced recovery after surgery (ERAS) for spine surgery: a systematic review. World Neurosurg 2019; 130: 415–426. 31276851 10.1016/j.wneu.2019.06.181

[15] Levett DZH Grimmett C . Psychological factors, prehabilitation and surgical outcomes: evidence and future directions. Anaesthesia 2019; 74(Suppl 1): 36–42. 30604423 10.1111/anae.14507

[16] Kleykamp BA Ferguson MC McNicol E , et al. The prevalence of psychiatric and chronic pain comorbidities in fibromyalgia: an ACTTION systematic review. Semin Arthritis Rheum 2021; 51: 166–174. 33383293 10.1016/j.semarthrit.2020.10.006

[17] Wynter-Blyth V Moorthy K . Prehabilitation: preparing patients for surgery. BMJ 2017; 358: j3702. 28790033 10.1136/bmj.j3702

[18] Mullins CF Bak B Moore D . Pre-outpatient group education and assessment in chronic pain: a systematic review. Pain Med 2022; 23: 89–104. 33787896 10.1093/pm/pnab036

[19] Killingback C Thompson M Chipperfield S , et al. Physiotherapists’ views on their role in self-management approaches: a qualitative systematic review. Physiother Theory Pract 2022; 38: 2134–2148. 33813990 10.1080/09593985.2021.1911011

[20] Lorig KR Holman H . Self-management education: history, definition, outcomes, and mechanisms. Ann Behav Med 2003; 26: 1–7. 12867348 10.1207/S15324796ABM2601_01

[21] Higueras A Gonzalez G de Lourdes Bolaños M , et al. Economic impact of the implementation of an enhanced recovery after surgery (ERAS) protocol in a bariatric patient undergoing a roux-en-Y gastric bypass. Int J Environ Res Publ Health 2022; 19: 14946. 10.3390/ijerph192214946 PMC9690327 36429661

[22] Duarte R Lloyd A Kotas E , et al. Are acceptance and mindfulness-based interventions ‘value for money’? Evidence from a systematic literature review. Br J Clin Psychol 2019; 58: 187–210. 30499217 10.1111/bjc.12208 PMC6588093

[23] Bicego A Monseur J Rousseaux F , et al. Drop-out from chronic pain treatment programmes: is randomization justified in biopsychosocial approaches? J Rehabil Med 2021; 53: jrm00185. 33829274 10.2340/16501977-2824 PMC8814856

[24] Gillis C Gramlich L Culos-Reed SN , et al. Third-variable effects: tools to understand who, when, why, and how patients benefit from surgical prehabilitation. J Surg Res 2021; 258: 443–452. 33129504 10.1016/j.jss.2020.09.026

[25] Slovinec D’Angelo ME Pelletier LG Reid RD , et al. The roles of self-efficacy and motivation in the prediction of short- and long-term adherence to exercise among patients with coronary heart disease. Health Psychol 2014; 33: 1344–1353. 25133848 10.1037/hea0000094

[26] Huijs E Stigt BJ Roos ND , et al. The feasibility of an anti-inflammatory diet in endometriosis: barriers and facilitators perceived by endometriosis patients. Reprod Biomed Online 2024; 48: 103624. DOI: 10.1016/j.rbmo.2023.103624. 38181648

[27] Louw A Diener I Landers M , et al. Preoperative pain neuroscience education for lumbar radiculopathy: a multi-center randomized controlled trial with one-year follow-up. Spine 1986; 39: 1449–1457. 10.1097/BRS.0000000000000444 24875964

[28] Burgess LC Arundel J Wainwright TW . The effect of preoperative education on psychological, clinical and economic outcomes in elective spinal surgery: a systematic review. Healthcare 2019; 7: 48. 30901875 10.3390/healthcare7010048 PMC6473918

[29] Hartley M Neubrander J Repede E . Evidence-based spine preoperative education. Int J Orthop Trauma Nurs 2012; 16: 65–75.

[30] Boukili IE Flaris AN Mercier F , et al. Prehabilitation before major abdominal surgery: evaluation of the impact of a perioperative clinical pathway, a pilot study. Scand J Surg 2022; 111: 14574969221083394. 35437086 10.1177/14574969221083394

[31] Fulop A Lakatos L Susztak N , et al. The effect of trimodal prehabilitation on the physical and psychological health of patients undergoing colorectal surgery: a randomised clinical trial. Anaesthesia 2021; 76: 82–90. 32761611 10.1111/anae.15215

[32] WHO . COVID-19 has caused major disruptions and backlogs in health care, new WHO study finds. Geneva: WHO, 2024. https://www.who.int/europe/news/item/20-07-2022-covid-19-has-caused-major-disruptions-and-backlogs-in-health-care--new-who-study-finds (accessed 30 October 2023).

[33] Deslauriers S Roy J-S Bernatsky S , et al. Factors associated with waiting times for persons with rheumatic conditions in multidisciplinary pain treatment facilities. J Pain Res 2019; 12: 2379–2390. 31534361 10.2147/JPR.S206519 PMC6681557

[34] Connelly D. Some patients with chronic pain face waiting years to see a specialist. Pharmaceut J, 2020. https://pharmaceutical-journal.com/article/news/some-patients-with-chronic-pain-face-waiting-years-to-see-a-specialist (accessed 30 October 2023).

[35] Csupak B Sommer JL Jacobsohn E , et al. A population-based examination of the co-occurrence and functional correlates of chronic pain and generalized anxiety disorder. J Anxiety Disord 2018; 56: 74–80. 29703452 10.1016/j.janxdis.2018.04.005

[36] Kroenke K Outcalt S Krebs E , et al. Association between anxiety, health-related quality of life and functional impairment in primary care patients with chronic pain. Gen Hosp Psychiatr 2013; 35: 359–365. 10.1016/j.genhosppsych.2013.03.020 23639186

[37] Racine M . Chronic pain and suicide risk: a comprehensive review. Prog Neuro-Psychopharmacol Biol Psychiatry 2018; 87: 269–280. 10.1016/j.pnpbp.2017.08.020 28847525

[38] Carr T Teucher U Casson AG . Waiting for scheduled surgery: a complex patient experience. J Health Psychol 2017; 22: 290–301. 26349617 10.1177/1359105315603464

[39] Bourke MJ Ferguson D Cooke M . Patient experiences of self-management for chronic low back pain: a qualitative study. Phys Ther 2022; 102: pzac030. 35358311 10.1093/ptj/pzac030

[40] Deslauriers S Roy J-S Bernatsky S , et al. The burden of waiting to access pain clinic services: perceptions and experiences of patients with rheumatic conditions. BMC Health Serv Res 2021; 21: 160. 33602224 10.1186/s12913-021-06114-y PMC7891805

[41] Lynch ME Williamson OD Banfield JC . COVID-19 impact and response by Canadian pain clinics: a national survey of adult pain clinics. Can J Pain 2020; 4: 204–209. 33987499 10.1080/24740527.2020.1783218 PMC7951169

[42] Wasserman D Iosue M Wuestefeld A , et al. Adaptation of evidence-based suicide prevention strategies during and after the COVID-19 pandemic. World Psychiatr 2020; 19: 294–306. 10.1002/wps.20801 PMC7491639 32931107

[43] Hruschak V Flowers KM Azizoddin DR , et al. Cross-sectional study of psychosocial and pain-related variables among patients with chronic pain during a time of social distancing imposed by the coronavirus disease 2019 pandemic. Pain 2021; 162: 619. 33230007 10.1097/j.pain.0000000000002128 PMC7808279

[44] Hadi MA Alldred DP Briggs M , et al. Treated as a number, not treated as a person’: a qualitative exploration of the perceived barriers to effective pain management of patients with chronic pain. BMJ Open 2017; 7: e016454. 10.1136/bmjopen-2017-016454 PMC5541634 28606909

[45] Michie S van Stralen MM West R . The behaviour change wheel: a new method for characterising and designing behaviour change interventions. Implement Sci 2011; 6: 42. 21513547 10.1186/1748-5908-6-42 PMC3096582

[46] Álvarez-Rodríguez J Leirós-Rodríguez R Morera-Balaguer J , et al. The influence of the locus of control construct on the efficacy of physiotherapy treatments in patients with chronic pain: a systematic review. J Personalized Med 2022; 12: 232. 10.3390/jpm12020232 PMC8880621 35207720

[47] Musich S Wang SS Slindee L , et al. The association of pain locus of control with pain outcomes among older adults. Geriatr Nurs 2020; 41: 521–529. 31078323 10.1016/j.gerinurse.2019.04.005

[48] Müßgens D Burgard LC Kleine-Borgmann J , et al. Impact of the COVID-19 pandemic on patients with chronic pain in Germany: associations with expectations and control beliefs. Eur J Pain 2022; 26: 1343–1354. 35445510 10.1002/ejp.1955 PMC9087415

[49] Faculty of Pain Medicine of the Royal College of Anaesthetists . Core standards for pain management services in the UK. 2nd ed. London: Faculty of Pain Medicine of the Royal College of Anaesthetists, 2021. https://fpm.ac.uk/sites/fpm/files/documents/2021-07/FPM-Core-Standards-2021_1.pdf (accessed 25 January 2024).

[50] The British Pain Society . Guidelines for pain management programmes for adults. An evidence-based review prepared on behalf of The British Pain Society. London: The British Pain Society, 2021. https://www.britishpainsociety.org/static/uploads/resources/files/PMP_guidelines_8QD8FJF.pdf (accessed 25 January 2024).

[51] Bishop MD Bialosky JE Cleland JA . Patient expectations of benefit from common interventions for low back pain and effects on outcome: secondary analysis of a clinical trial of manual therapy interventions. J Man Manip Ther 2011; 19: 20–25. 22294850 10.1179/106698110X12804993426929 PMC3172953

[52] Cormier S Lavigne GL Choinière M , et al. Expectations predict chronic pain treatment outcomes. Pain 2016; 157: 329–338. 26447703 10.1097/j.pain.0000000000000379

[53] Haanstra TM Kamper SJ Williams CM , et al. Does adherence to treatment mediate the relationship between patients’ treatment outcome expectancies and the outcomes of pain intensity and recovery from acute low back pain? Pain 2015; 156: 1530–1536. 25906348 10.1097/j.pain.0000000000000198

[54] Bingel U . Placebo 2.0: the impact of expectations on analgesic treatment outcome. Pain 2020; 161(Suppl 1): S48–S56. 33090739 10.1097/j.pain.0000000000001981

[55] Bingel U Wanigasekera V Wiech K , et al. The effect of treatment expectation on drug efficacy: imaging the analgesic benefit of the opioid remifentanil. Sci Transl Med 2011; 3: 70ra14. 10.1126/scitranslmed.3001244 21325618

[56] Mohamed Mohamed WJ Joseph L Canby G , et al. Are patient expectations associated with treatment outcomes in individuals with chronic low back pain? a systematic review of randomised controlled trials. Int J Clin Pract 2020; 74: e13680. 33166045 10.1111/ijcp.13680

[57] Broderick JE Keefe FJ Schneider S , et al. Cognitive behavioral therapy for chronic pain is effective, but for whom? Pain 2016; 157: 2115–2123. 27227692 10.1097/j.pain.0000000000000626

[58] Simm R Barker C . Five years of a community pain service solution-focused pain management programme: extended data and reflections. Br J Pain 2018; 12: 113–121. 29796263 10.1177/2049463717744358 PMC5958514

[59] Dilgul M McNamee P Orfanos S , et al. Why do psychiatric patients attend or not attend treatment groups in the community: a qualitative study. PLoS One 2018; 13: e0208448. 30543646 10.1371/journal.pone.0208448 PMC6292613

[60] Galvin BM Randel AE Collins BJ , et al. Changing the focus of locus (of control): a targeted review of the locus of control literature and agenda for future research. J Organ Behav 2018; 39: 820–833.

[61] Osborne RH Batterham R Livingston J . The evaluation of chronic disease self-management support across settings: the international experience of the health education impact questionnaire quality monitoring system. Nurs Clin 2011; 46: 255–270. 10.1016/j.cnur.2011.05.010 21791261

[62] Wallston KA Wallston BS DeVellis R . Development of the multidimensional health locus of control (MHLC) scales. Health Educ Monogr 1978; 6: 160–170. 689890 10.1177/109019817800600107

[63] Härkäpää K Järvikoski A Mellin G , et al. Health locus of control beliefs and psychological distress as predictors for treatment outcome in low-back pain patients: results of a 3-month follow-up of a controlled intervention study. Pain 1991; 46: 35–41. 1832753 10.1016/0304-3959(91)90031-R

[64] Michie S West R Finnerty AN , et al. Representation of behaviour change interventions and their evaluation: development of the upper level of the behaviour change intervention Ontology. Wellcome Open Res 2020; 5: 123. 33614976 10.12688/wellcomeopenres.15902.1 PMC7868854

[65] Wahl AK Opseth G Nolte S , et al. Is regular use of physiotherapy treatment associated with health locus of control and self-management competency? a study of patients with musculoskeletal disorders undergoing physiotherapy in primary health care. Musculoskelet Sci Pract 2018; 36: 43–47. 29729545 10.1016/j.msksp.2018.04.008

[66] Zuercher-Huerlimann E Stewart JA Egloff N , et al. Internal health locus of control as a predictor of pain reduction in multidisciplinary inpatient treatment for chronic pain: a retrospective study. J Pain Res 2019; 12: 2095–2099. 31372026 10.2147/JPR.S189442 PMC6626892

[67] Vergara F Rosa J Orozco C , et al. Evaluation of learned helplessness, self-efficacy and disease activity, functional capacity and pain in Argentinian patients with rheumatoid arthritis. Scand J Rheumatol 2017; 46: 17–21. 27095187 10.3109/03009742.2016.1155643

[68] Keedy NH Keffala VJ Altmaier EM , et al. Health locus of control and self-efficacy predict back pain rehabilitation outcomes. Iowa Orthop J 2014; 34: 158–165. 25328476 PMC4127740

[69] Benyon K Hill S Zadurian N , et al. Coping strategies and self-efficacy as predictors of outcome in osteoarthritis: a systematic review. Muscoskel Care 2010; 8: 224–236. 10.1002/msc.187 20963846

[70] Karasawa Y Yamada K Iseki M , et al. Association between change in self-efficacy and reduction in disability among patients with chronic pain. PLoS One 2019; 14: e0215404. 30990842 10.1371/journal.pone.0215404 PMC6467389

[71] Martinez-Calderon J Zamora-Campos C Navarro-Ledesma S , et al. The role of self-efficacy on the prognosis of chronic musculoskeletal pain: a systematic review. J Pain 2018; 19: 10–34. 28939015 10.1016/j.jpain.2017.08.008

[72] Wilson M Roll JM Corbett C , et al. Empowering patients with persistent pain using an internet-based self-management program. Pain Manag Nurs 2015; 16: 503–514. 26088940 10.1016/j.pmn.2014.09.009

[73] Martinez-Calderon J Meeus M Struyf F , et al. The role of self-efficacy in pain intensity, function, psychological factors, health behaviors, and quality of life in people with rheumatoid arthritis: a systematic review. Physiother Theory Pract 2020; 36: 21–37. 29873569 10.1080/09593985.2018.1482512

[74] Cheng S-T Leung CMC Chan KL , et al. The relationship of self-efficacy to catastrophizing and depressive symptoms in community-dwelling older adults with chronic pain: a moderated mediation model. PLoS One 2018; 13: e0203964. 30226892 10.1371/journal.pone.0203964 PMC6143242

[75] Damush Tm. Kroenke K Bair Mj. , et al. Pain self-management training increases self-efficacy, self-management behaviours and pain and depression outcomes. Eur J Pain 2016; 20: 1070–1078. 26849410 10.1002/ejp.830

[76] Elbers S Wittink H Pool JJM , et al. The effectiveness of generic self-management interventions for patients with chronic musculoskeletal pain on physical function, self-efficacy, pain intensity and physical activity: a systematic review and meta-analysis. Eur J Pain 2018; 22: 1577–1596. 29845678 10.1002/ejp.1253 PMC6175326

[77] Ndosi M Johnson D Young T , et al. Effects of needs-based patient education on self-efficacy and health outcomes in people with rheumatoid arthritis: a multicentre, single blind, randomised controlled trial. Ann Rheum Dis 2016; 75: 1126–1132. 26162769 10.1136/annrheumdis-2014-207171 PMC4893097

[78] Martinez-Calderon J Struyf F Meeus M , et al. The association between pain beliefs and pain intensity and/or disability in people with shoulder pain: a systematic review. Musculoskelet Sci Pract 2018; 37: 29–57. 29980139 10.1016/j.msksp.2018.06.010

[79] Wilson JM Schreiber KL Mackey S , et al. Increased pain catastrophizing longitudinally predicts worsened pain severity and interference in patients with chronic pain and cancer: a collaborative health outcomes information registry study (CHOIR). Psycho Oncol 2022; 31: 1753–1761. 10.1002/pon.6020 PMC9910323 35988161

[80] Sullivan MJL Bishop SR Pivik J . The pain catastrophizing scale: development and validation. Psychol Assess 1995; 7: 524–532.

[81] Hardman R Lawn S Tsourtos G . Pain self-management: easier said than done? factors associated with early dropout from pain self-management in a rural primary care population. Pain Med 2019; 20: 267–277. 30203053 10.1093/pm/pny167

[82] Oosterhaven J Wittink H Dekker J , et al. Pain catastrophizing predicts dropout of patients from an interdisciplinary chronic pain management programme: a prospective cohort study. J Rehabil Med 2019; 51: 761–769. 31544215 10.2340/16501977-2609

[83] Biller N Arnstein P Caudill MA , et al. Predicting completion of a cognitive-behavioral pain management program by initial measures of a chronic pain patient’ s readiness for change. Clin J Pain 2000; 16: 352–359. 11153793 10.1097/00002508-200012000-00013

[84] Craner JR Sperry JA Evans MM . The relationship between pain catastrophizing and outcomes of a 3-week comprehensive pain rehabilitation program. Pain Med 2016; 17: 2026–2035. 27230076 10.1093/pm/pnw070

[85] Miller J MacDermid JC Walton DM , et al. Chronic pain self-management support with pain science education and exercise (commence) for people with chronic pain and multiple comorbidities: a randomized controlled trial. Arch Phys Med Rehabil 2020; 101: 750–761. 32004517 10.1016/j.apmr.2019.12.016

[86] Burns JW Day MA Thorn BE . Is reduction in pain catastrophizing a therapeutic mechanism specific to cognitive-behavioral therapy for chronic pain? Transl Behav Med 2012; 2: 22–29. 24073095 10.1007/s13142-011-0086-3 PMC3717814

[87] Scott EL Kroenke K Wu J , et al. Beneficial effects of improvement in depression, pain catastrophizing, and anxiety on pain outcomes: a 12-month longitudinal analysis. J Pain 2016; 17: 215–222. 26542153 10.1016/j.jpain.2015.10.011

[88] Shim E-J Hahm B-J Go DJ , et al. Modeling quality of life in patients with rheumatic diseases: the role of pain catastrophizing, fear-avoidance beliefs, physical disability, and depression. Disabil Rehabil 2018; 40: 1509–1516. 28291952 10.1080/09638288.2017.1300691

[89] Xu J Twiggs J Parker D , et al. The association between anxiety, depression, and locus of control with patient outcomes following total knee arthroplasty. J Arthroplasty 2020; 35: 720–724. 31708293 10.1016/j.arth.2019.10.022

[90] IASP. Personalised Care for Low Back Pain. International Association for the Study of Pain (IASP) , 2011. https://www.iasp-pain.org/resources/fact-sheets/personalised-care-for-low-back-pain/, (accessed 26 October 2022).

[91] Hall JA Jowett S Lewis M , et al. The STarT back stratified care model for nonspecific low back pain: a model-based evaluation of long-term cost-effectiveness. Pain 2021; 162: 702. 32868748 10.1097/j.pain.0000000000002057

[92] NICE . Quality statement 4: individualised care | Patient experience in adult NHS services | Quality standards. Ra’anana: NICE, 2012. https://www.nice.org.uk/guidance/qs15/chapter/quality-statement-4-individualised-care (accessed 30 October 2023).

[93] Lim YZ Chou L Au RT , et al. People with low back pain want clear, consistent and personalised information on prognosis, treatment options and self-management strategies: a systematic review. J Physiother 2019; 65: 124–135. 31227280 10.1016/j.jphys.2019.05.010

[94] Chavez JL Porucznik CA Gren LH , et al. The impact of preoperative mindfulness-based stress reduction on postoperative outcomes in lumbar spine degenerative disease: 3-month and 12-month results of a pilot study. World Neurosurg 2020; 139: e230–e236. 32278820 10.1016/j.wneu.2020.03.186

[95] Shah N Costello K Mehta A , et al. Applications of digital health technologies in knee osteoarthritis: narrative review. JMIR Rehabil Assist Technol 2022; 9: e33489. 35675102 10.2196/33489 PMC9218886

[96] Yi JL Porucznik CA Gren LH , et al. The impact of preoperative mindfulness-based stress reduction on postoperative patient-reported pain, disability, quality of life, and prescription opioid use in lumbar spine degenerative disease: a pilot study. World Neurosurg 2019; 121: e786–e791. 30312812 10.1016/j.wneu.2018.09.223

[97] Rini C Porter LS Somers TJ , et al. Automated Internet-based pain coping skills training to manage osteoarthritis pain: a randomized controlled trial. Pain 2015; 156: 837–848. 25734997 10.1097/j.pain.0000000000000121 PMC4402249

[98] Schmidt M Eckardt R Scholtz K , et al. Patient empowerment improved perioperative quality of care in cancer patients aged ≥ 65 years – a randomized controlled trial. PLoS One 2015; 10: e0137824. 26378939 10.1371/journal.pone.0137824 PMC4574984

[99] Fraval A Chandrananth J Chong YM , et al. Internet based patient education improves informed consent for elective orthopaedic surgery: a randomized controlled trial. BMC Muscoskel Disord 2015; 16: 14. 10.1186/s12891-015-0466-9 PMC4331305 25885962

[100] Timmers T Janssen L van der Weegen W , et al. The effect of an app for day-to-day postoperative care education on patients with total knee replacement: randomized controlled trial. JMIR MHealth UHealth 2019; 7: e15323. 31638594 10.2196/15323 PMC6914303

[101] Timmers T Janssen L Pronk Y , et al. Assessing the efficacy of an educational smartphone or tablet app with subdivided and interactive content to increase patients’ medical knowledge: randomized controlled trial. JMIR MHealth UHealth 2018; 6: e10742. 30578185 10.2196/10742 PMC6320423

[102] Keogh A Tully MA Matthews J , et al. A review of behaviour change theories and techniques used in group based self-management programmes for chronic low back pain and arthritis. Man Ther 2015; 20: 727–735. 25865062 10.1016/j.math.2015.03.014

[103] McMannus J Constable M Bunten A , et al. Improving people’s health: applying behavioural and social sciences. London, UK: Public Health EnglandGOV. 2018. https://www.gov.uk/government/publications/improving-peoples-health-applying-behavioural-and-social-sciences (accessed 30 October 2023).

[104] Stacey FG James EL Chapman K , et al. A systematic review and meta-analysis of social cognitive theory-based physical activity and/or nutrition behavior change interventions for cancer survivors. J Cancer Surviv 2015; 9: 305–338. 25432633 10.1007/s11764-014-0413-z PMC4441740

[105] NICE . Patient Public Involvement Policy. National Institute for Health and Care Excellence. 2021. https://www.nice.org.uk/about/nice-communities/nice-and-the-public/public-involvement/public-involvement-programme/patient-public-involvement-policy (accessed 30 October 2023).

[106] Willett M Duda J Fenton S , et al. Effectiveness of behaviour change techniques in physiotherapy interventions to promote physical activity adherence in lower limb osteoarthritis patients: a systematic review. PLoS One 2019; 14: e0219482. 31291326 10.1371/journal.pone.0219482 PMC6619772

[107] Marques MM Wright AJ Corker E , et al. The Behaviour Change Technique Ontology: Transforming the Behaviour Change Taxonomy V1. Wellcome Open Research. 2023; 8: 308–325. 37593567 10.12688/wellcomeopenres.19363.2 PMC10427801

[108] McCracken LM . Personalized pain management: is it time for process-based therapy for particular people with chronic pain? Eur J Pain 2023; 27: 1044–1055. 36755478 10.1002/ejp.2091

[109] Schumann ME Coombes BJ Gascho KE , et al. Pain catastrophizing and pain self-efficacy mediate interdisciplinary pain rehabilitation program outcomes at posttreatment and follow-up. Pain Med 2022; 23: 697–706. 34519826 10.1093/pm/pnab271

